# TRAV7-2*02 Expressing CD8^+^ T Cells Are Responsible for Palladium Allergy

**DOI:** 10.3390/ijms18061162

**Published:** 2017-05-31

**Authors:** Yuri Takeda, Yoshiko Suto, Koyu Ito, Wataru Hashimoto, Tadashi Nishiya, Kyosuke Ueda, Takayuki Narushima, Tetsu Takahashi, Kouetsu Ogasawara

**Affiliations:** 1 Department of Immunobiology, Institute of Development, Aging and Cancer, Tohoku University 4-1 Seiryo-machi, Aoba-ku, Sendai 980-8575, Japan; yuri.takeda@dent.tohoku.ac.jp (Y.T.); yosshi.snoopy.11r25@gmail.com (Y.S.); Koyu.ito.c6@tohoku.ac.jp (K.I.); 2 Department of Oral and Maxillofacial Surgery, Graduate School of Dentistry, Tohoku University, 4-1 Seiryo-machi, Aoba-ku, Sendai, Miyagi 980-8575, Japan; wataru-thk@umin.ac.jp (W.H.); tetsu@dent.tohoku.ac.jp (T.T.); 3 Department of Pharmacology, School of Pharmaceutical Sciences, Ohu University, 31-1 Misumido, Tomitamachi, Koriyama, Fukushima 963-8611, Japan; t-nishiya@pha.ohu-u.ac.jp; 4 Department of Materials Processing, Graduate School of Engineering, Tohoku University, 6-6-02 Aza Aoba, Aramaki, Aoba-ku, Sendai, Miyagi 980-8579, Japan; ueda@material.tohoku.ac.jp (K.U.); narut@material.tohoku.ac.jp (T.N.)

**Keywords:** animal models, autoimmunity, T cells, biomaterial(s), metal allergy, T cell receptor (TCR)

## Abstract

While metallic biomaterials have led to an improvement in the quality of life, metal allergies, especially to palladium (Pd), has caused a recent increase in allergic patients. Metal allergy is known to be a T cell-mediated delayed-type hypersensitivity (DTH); however, the pathogenic T cell subsets and the specific T cell receptor (TCR) have not been identified. Therefore, we attempted to identify the pathogenic T cells responsible for Pd allergy. We found that activating CD8^+^ T cells significantly increased and that the TRAV (TCRα variable) 7-2*02 chain skewed in Pd allergic mice. Furthermore, adoptive transfer experiments revealed that in vitro-cultured Pd-stimulated antigen presenting cells (APCs) function as memory APCs with recipient mice developing Pd allergy and that the frequency of TRAV7-2*02 increases the same as conventional Pd allergic mice. In contrast, neither proliferation of CD8^+^ T cells nor increasing of TRAV7-2*02 was observed in major histocompatibility complex I (MHC I)-deficient Pd-APCs transferred to mice. Taken together, we revealed that TRAV7-2*02-expressing CD8^+^ T cells are the pathogenic T cells for the development of Pd allergy. We also identified the CDR3 consensus motif of pathogenic TCRs as CAAXSGSWQLIF in TRAV7-2*02/TRAJ (TCRα junction)22*01 positive cells. These results suggest that the specific TCRs represent novel targets for the development of diagnostics and treatments for metal allergy.

## 1. Introduction

Metal is superior to other materials in hardness, strength, durability, and workability and, therefore, is broadly useful in many fields. Palladium (Pd), in particular, has a wide array of uses, including as an extremely important metallic biomaterial for dentistry in the reconstruction of occlusions during dental restorations. However, metallic biomaterials sometimes cause metal allergy [1], and the incidence of Pd allergy, in particular, has increased recently [2]. However, the pathological mechanism of metal allergy has not been clarified. Clinical symptoms of metal allergy include intractable diseases, such as dermatitis, inflammation of oral mucosa, oral lichen planus, and stomatitis.

Metal allergy is categorized as a delayed-type hypersensitivity (DTH) reaction, which is a T cell-mediated disease with onset at 24 hours after exposure to the causal metal [3,4]. The pathogenic mechanism of metal allergy includes the function of metal ions as haptens that exert antigenicity and induce pathogenic T cells, which cause metal allergy. T cells are classified into two broad subsets, CD4^+^ T cells and CD8^+^ T cells. CD4^+^ T cells binding major histocompatibility complex II (MHC II) on presenting cells and play roles as helper and regulatory T cells by producing cytokines. CD8^+^ T cells recognize MHC I and function as proinflammatory T cells that directly kill infected or transformed cells. It has been reported that both CD8^+^ and CD4^+^ T cells are required for DTH [3]. Thus, both types of cells [5,6] were generally thought to be responsible for Pd allergy; however, controversy remains regarding the role of pathogenic T cell subsets in DTH.

Various cytokines control the activation or inhibition of immune responses that comprise the host biological defense system against foreign antigens. IFN-γ, the most important cytokine in metal allergy, is an inflammatory cytokine that enhances immune responses, including Th1 responses and cytotoxic activity [6,7,8]. Previously, we reported that there was a decrease in ear swelling in IFN-γ-deficient mice (IFN-γ^−/−^ mice) compared to wild-type (WT) mice after Pd challenge [6]; however, in a previous study the distribution of T cell subsets was not examined in IFN-γ^−/−^ mice after Pd challenge.

In order to establish acquired immunity, the TCR recognizes antigenic peptides present in the context of MHC, which leads to T cell activation [9,10]. To obtain vast repertoire diversity, the TCR α and β genes undergo complex rearrangement of variable (V), diverse (D), and junction (J) region gene segments resulting in the generation of a specific TCR from 1 × 10^18^ possible clonotypes. Examination of peripheral blood mononuclear cells obtained from patients with metal allergy showed that metal ions can induce proliferation of human T cells in vitro and limited TCRs are utilized in human T cells from metal allergy patients [11]. However, the specific TCR present on pathogenic T cells of patients with Pd allergy has not previously been identified. Therefore, we attempted to identify the T cell subsets responsible for Pd allergy and investigated whether a specific TCR exists on these pathogenic T cells.

## 2. Results

### 2.1. Activated T Cells Accumulate in Regional Lymph Nodes during Pd Allergy

**Figure 1:**
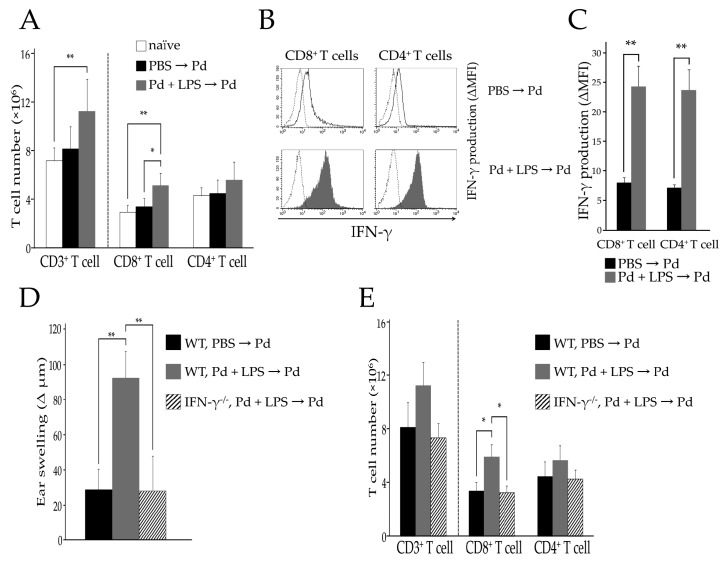
Number of T cells in each subset (**A**,**E**), IFN-γ production (**B**,**C**), and ear swelling (**D**) are represented at 24 h post-Pd challenge among naïve, unsensitized-WT (WT, PBS → Pd), sensitized-WT (WT, Pd + LPS → Pd), and sensitized-IFN-γ^−/−^ mice (WT, Pd + LPS → Pd), (*n* = 4–5). Values are means ± Standard deviation (SD). * *p* < 0.05 and ** *p* < 0.01. Similar results were obtained in two independent experiments.

To examine the accumulation of activated T cells in regional lymph nodes, we analyzed the T cell populations present in submandibular lymph node (SLN) cells of Pd-allergic mice by flow cytometry. To this end, Pd + lipopolysaccharide (LPS)-sensitized WT mice (sensitized-WT mice) and phosphate buffered saline (PBS)-injected WT mice (unsensitized-WT mice) were challenged by injection of Pd solution into ear auricles and ear swelling was assessed (Figure S1A). Following Pd challenge, ear swelling of sensitized-WT mice was significantly increased compared to that of unsensitized-WT mice (** *p* < 0.01). Moreover, WT mice sensitized with Pd alone or LPS alone and then challenged with Pd showed no evidence of ear swelling [6]. Both CD8^+^ and CD4^+^ T cells accumulated in ear auricles of sensitized WT mice (Figure S1B). Since the ears were most swollen at 24 h post Pd challenge, we assessed the T cell population in the SLNs at this time and found no difference in CD3^+^ T cell numbers between unsensitized-WT mice and naïve mice. In contrast, there was a significant increase in CD3^+^ T cells in the SLNs of sensitized-WT mice (** *p* < 0.01) (Figure 1A). Although CD4^+^ T cells appeared to be slightly increased in the SLN of sensitized WT mice, this difference was not statistically significant (Figure 1A). In contrast, CD8^+^ T cells were significantly increased in the SLN of sensitized WT mice compared to unsensitized WT (* *p* < 0.05) or naïve mice (** *p* < 0.01) (Figure 1A). We next measured IFN-γ production by SLN T cells. Previously, we showed that IFN-γ producing CD8^+^ T cells are responsible for Pd allergy [6]. However, in the previous study we analyzed IFN-γ production in the limited condition of T cells enriched by sequential adoptive transfer in Pd allergic mice. Therefore, we investigated which T cells are activated in a conventional model of Pd allergy. Interestingly, not only CD8^+^ T cells, but also CD4^+^ T cells produced IFN-γ in sensitized-WT mice (** *p* < 0.01) (Figure 1B,C). Since ear auricles were not swollen in response to Pd challenge of IFN-γ^−/−^ mice (Figure 1D), we assessed CD8^+^ and CD4^+^ T cells in the SLN cells of sensitized-IFN-γ^−/−^ mice 24 h after Pd challenge and found no increase in CD8^+^ T cells (* *p* < 0.05) compared to that seen in sensitized-WT mice (Figure 1E). These results suggest that activation of CD8^+^ T cells, rather than CD4^+^ T cells, enhances the development of Pd allergy.

### 2.2. The Pathogenic TCR Repertoire in Pd Allergic Mice

**Figure 2:**
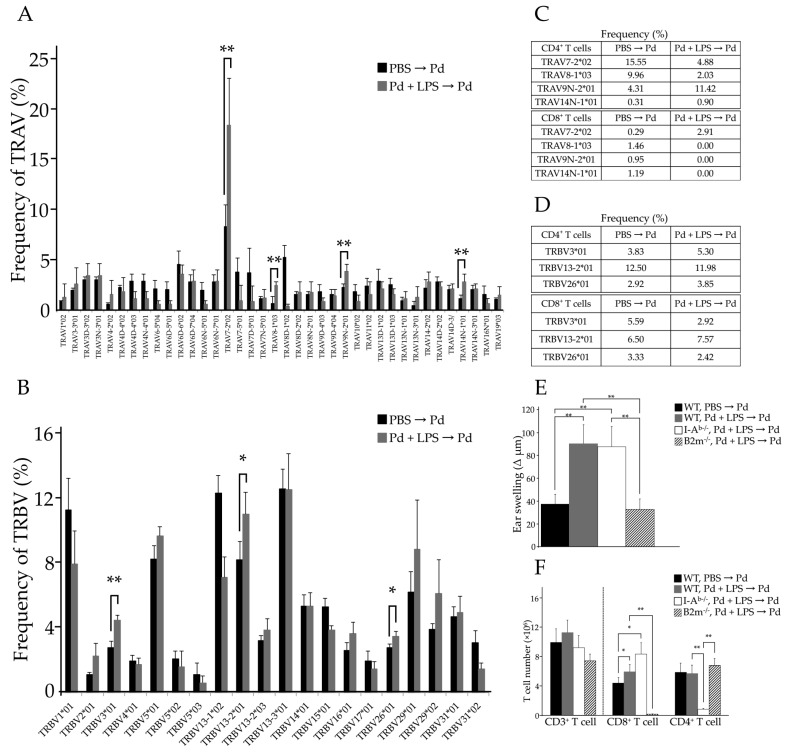
The frequency of TRAV (**A**) and TRBV (**B**) repertoires were compared between unsensitized-WT mice and sensitized-WT mice (*n* = 4); (**C**,**D**) The skewing of TCR repertoires in CD8^+^ and CD4^+^ T cells was analyzed. Ear swelling (**E**) and the number of T cells in each subset (**F**) were compared among unsensitized-WT mice, sensitized-WT mice, sensitized-I-A^b−/−^ mice, and sensitized-B2m^−/−^ mice (*n* = 4–5). Values are means ± SD. * *p* < 0.05 and ** *p* < 0.01. Similar results of ear swelling (**E**) and T cell numbers (**F**) were obtained in two independent experiments.

To identify the specific TCRs that contribute to Pd allergy, we sequenced the entire TCR repertoire present in the SLNs of Pd allergic mice. For this, SLN cells were collected 24 h after Pd challenge and the TCRα and TCRβ chain repertoires were examined. The results showed a clear skewing of TCR repertoires in SLN cells of sensitized-WT mice (Figure 2A,B). These repertoires were selected with a greater than 1% frequency in sensitized-WT mice. For the TCRα chain, frequencies of TRAV7-2*02, TRAV8-1*03, TRAV9N-2*01, and TRAV14N-1*01 were significantly increased in sensitized-WT mice (** *p* < 0.01) compared to that in unsensitized WT mice, (Figure 2A). Interestingly, among all repertoires, the frequency of TRAV7-2*02 was found to be greatly increased. For the TCRβ chain, frequencies of TRBV (TCRβ variable) 3*01 (** *p* < 0.01), TRBV13-2*01 (* *p* < 0.05), and TRBV26*01 (* *p* < 0.05) were significantly increased in sensitized-WT mice (Figure 2B), and TRBV13-2*01 was the most frequently represented of the TRBV repertoire. To examine whether these specific TRAV and TRBV repertoires are expressed on both CD8^+^ and CD4^+^ T cells, we purified CD8^+^ and CD4^+^ T cells from SLN cells of Pd allergic mice and examined their TCR repertories (Figure S2). Although TRAV7-2*02, TRAV8-1*03, TRAV9N-2*01, and TRAV14N-1*01 TCRα chains were expressed on CD4^+^ T cells in sensitized-WT mice, only TRAV7-2*02 was expressed on CD8^+^ T cells (Figure 2C). Furthermore, the frequency of TRAV7-2*02 in sensitized-WT mice was 10 times higher than that in unsensitized-WT mice (Figure 2C). In addition, TRAV8-1*03, TRAV9N-2*01, and TRAV14N-1*01 were not detected on CD8^+^ T cells (Figure 2C). For the TCRβ chain, TRBV13-2*01 not found to be significantly increased on either CD4^+^ or CD8^+^ T cells in sensitized-WT mice compared to that in unsensitized-WT mice (Figure 2D). Thus, Pd allergy-specific TCRβ chain was not identified. These results suggest that the TCRα chain, but not the TCRβ chain, is skewed on CD8^+^ T cells in Pd allergy. Moreover, TRAV7-2*02 is likely the Pd allergy-specific TCR.

### 2.3. MHC I and Activated CD8^+^ T Cells Are Critical for Pd Allergy

To investigate whether MHC I or II plays a more important role in Pd allergy, we induced Pd allergy in C57BL/6-deficient β2m-microgloblin (B2m) mice (B2m^−/−^ mice) and C57BL/6-deficient MHC II *H2-Ab1* (I-A^b^) mice (I-A^b−/−^ mice). Since B2m constitutes a part of the MHC I molecule, MHC I is not expressed on the cell surface in these mice and, thus, CD8^+^ T cells are impaired, while in I-A^b−/−^ mice CD4^+^ T cells are impaired. Compared to unsensitized-WT mice, ear auricles were significantly more swollen in sensitized I-A^b−/−^ mice at 24 h after Pd challenge (** *p* < 0.01). However, ear swelling was not observed in B2m^−/−^ mice following Pd challenge (Figure 2E). In addition, CD4^+^ T cells were not significantly increased in SLNs of B2m^−/−^ mice (Figure 2F), while the number of CD8^+^ T cells in SLNs of I-A^b−/−^ mice was greatly increased (** *p* < 0.01) (Figure 2F). Furthermore, TRAV7-2*02 was highly expressed on SLN cells from Pd-sensitized I-A^b−/−^ and WT mice (data not shown). Therefore, MHC I is essential and activated CD8^+^ T cells are important for the development of Pd allergy.

### 2.4. Adoptive Transfer of In Vitro Prepared Pd-Treated APCs Induces Pd Allergy

**Figure 3:**
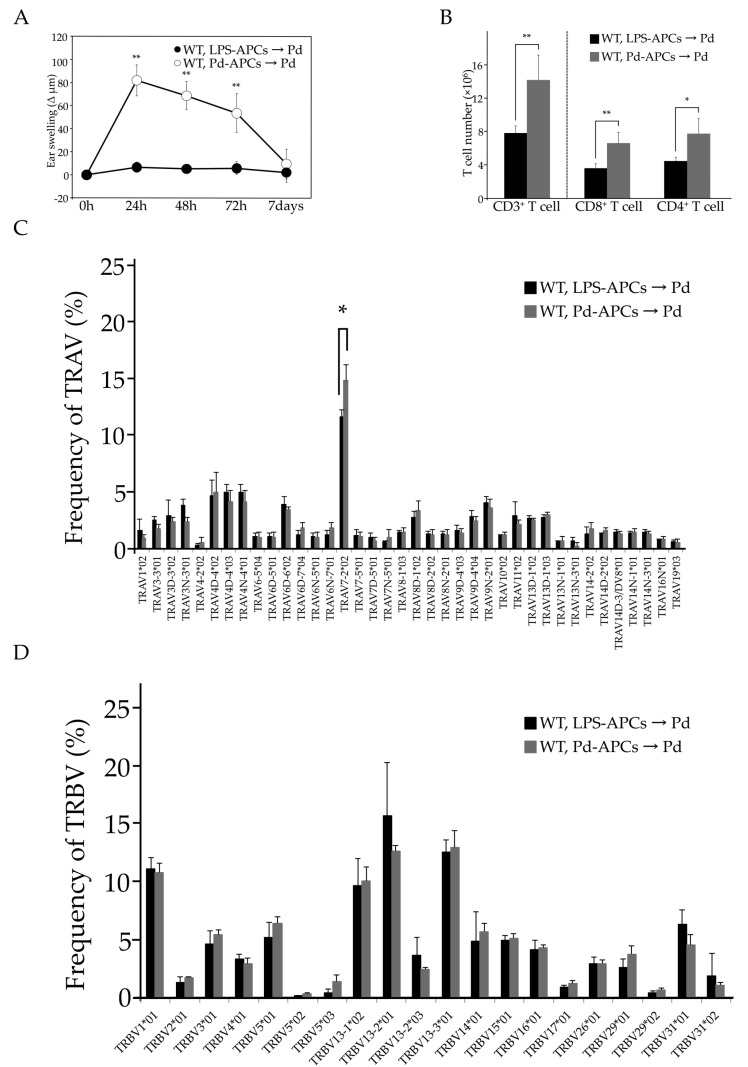
Ear swelling (**A**), number of T cells in each subset (**B**), TRAV (**C**), and TRBV (**D**) repertoires were examined after Pd challenge of mice given WT LPS-APCs or WT Pd-APCs (*n* = 4). Values are means ± SD. * *p* < 0.05 and ** *p* < 0.01. Similar results of ear swelling (**A**) and T cell numbers (**B**) were obtained in two independent experiments.

We examined whether Pd allergy could develop in mice with adoptively-transferred Pd-stimulated APCs. To this end, APCs were prepared from bone marrow cells and cultured in mouse macrophage colony stimulating factor (mM-CSF) containing medium. After seven days in culture, CD11b^+^ F4/80^+^ APCs were sorted and stimulated with Pd + LPS (Pd-APCs) or LPS (LPS-APCs) for 24 h. Flow cytometric analysis revealed that these APCs also express the co-stimulatory molecules CD80, CD86, and CD40 (Figure S3A). To assess their effects on Pd sensitization in mice, these APCs were adoptively transferred into naïve WT mice. Seven days after the transfer, recipient mice were challenged with Pd injection into ear auricles. Ear swelling in the mice given Pd-APCs was significantly increased (** *p* < 0.01) compared to that of mice given LPS-APCs (Figure 3A). Further, CD8^+^ and CD4^+^ T cells were significantly increased in SLNs of mice that received Pd-APCs (Figure 3B). These results indicate that APCs cultured with Pd + LPS in vitro function as the driving force in the development of Pd allergy. In addition, the TCR repertoire in mice given Pd-APCs is skewed in a manner similar to that seen in Pd-sensitized WT mice, with significant increases in TRAV7-2*02 expression, but not TRBV expression, starting 24 h after Pd challenge (Figure 3C,D). Therefore, Pd-memory APCs lead to Pd allergy, and the TCRα chain, rather than TCRβ chain, is involved in the development of Pd allergy.

### 2.5. MHC I-Dependent CD8^+^ T Cell Activation Is Essential for the Onset of Pd Allergy

**Figure 4:**
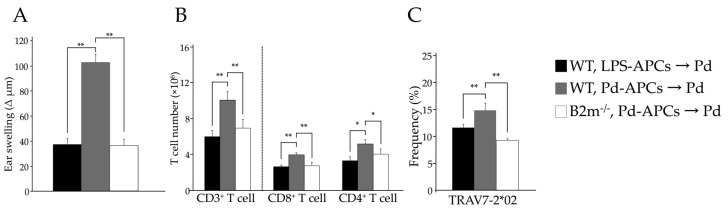
Ear thickness (**A**), number of cells in each T cell subset (**B**), and frequency of TRAV7-2*02 (**C**) were compared among mice given WT LPS-APCs, WT Pd-APCs, or B2m^−/−^ Pd-APCs (*n* = 4) followed by Pd challenge. Values are means ± SD. * *p* < 0.05 and ** *p* < 0.01. Similar results of (**A**,**B**) were obtained in two independent experiments.

Neither MHC I molecules or CD8^+^ T cells function in B2m^−/−^ mice as development of CD8^+^ T cells is impaired in the absence of this molecule. Thus, we investigated whether MHC I-deficient Pd-APCs derived from B2m^−/−^ mice are able to induce Pd allergy. To this end, we prepared WT Pd-APCs and B2m^−/−^ Pd-APCs and adoptively transferred them into naïve WT mice. Seven days later, these mice were challenged with Pd injection and ear thickness was measured from 24 h after the challenge. Ear swelling of B2m^−/−^ Pd-APC recipient mice was significantly less (** *p* < 0.01) than that of mice given WT Pd-APCs (Figure 4A). Neither CD4^+^ nor CD8^+^ T cells increased significantly in mice given B2m^−/−^ Pd-APCs compared to those given WT Pd-APCs (** *p* < 0.01) (Figure 4B). Moreover, the frequency of TRAV7-2*02 was not increased in mice given B2m^−/−^ Pd-APCs compared to mice given WT Pd-APCs (** *p* < 0.01) (Figure 4C). These results indicate that TRAV7-2*02 expressing CD8^+^ T cells are activated and proliferate in a manner dependent on MHC I, which leads to Pd allergy.

### 2.6. Identification of CDR3 Amino Acid Sequences in Pd Allergy

We assessed the skewing of J segment representation in TRAV7-2*02 during Pd allergy. Twenty-four hours from Pd challenge, TRAJ6*01, TRAJ15*01, TRAJ22*01, TRAJ26*01, TRAJ27*01, TRAJ31*01, and TRAJ37*01 within the TRAV7-2*02 repertoire were expressed in all Pd-sensitized WT mice (*n* = 4), with the frequency of TRAJ22*01 being markedly increased, comparatively (Table 1). CDR3 is known to be the hypervariable region that specifically recognizes the antigen-MHC complex [12,13]. Therefore, we also examined the CDR3 amino acid sequence of TRAV7-2*02/TRAJ22*01 and identified a consensus frame, CAAXSGSWQLIF (*X* = 1 to 4 amino acids), in all TRAV7-2*02/TRAJ22*01 (Table 2). In addition, the CDR3 amino acids common to TRAV7-2*02/TRAJ22*01 were expressed in Pd-APCs and transferred to mice at 24 h after Pd challenge (Table 3). Taken together, these results indicate that TRAV7-2*02/TRAJ22*01 is responsible for the induction of Pd allergy.

## 3. Discussion

In this study we demonstrate the skewing of specific TCRs during the development of Pd allergy and the contribution of CD8^+^ T cells, rather than CD4^+^ T cells, to the development of this allergy in mice. Moreover, we identify that TRAV7-2*02 expressing CD8^+^ T cells mainly function as pathogenic T cells. Furthermore, using adoptive transfer experiments we show that APCs stimulated in vitro with Pd function as metal memory APCs in vivo and induce sensitization for metal allergy when transferred. Indeed, skewing of TRAV7-2*02 was observed in the Pd allergic WT mice that received in vitro derived Pd-APCs. These results indicate that Pd memory APCs were generated in vitro and induce the pathogenic T cell activation seen during the Pd allergic response.

Although there has previously been controversy regarding this, we found that activated CD8^+^ T cells, rather than CD4^+^ T cells, play a key role as proinflammatory T cells in Pd allergy (Figure 1). Twenty-four hours after Pd challenge, both CD8^+^ and CD4^+^ T cells became activated and produced IFN-γ (Figure 1B,C). Thus, we assessed CD8^+^ and CD4^+^ T cells in the SLNs of sensitized-IFN-γ^−/−^ mice 24 hours after Pd challenge and found no increase in CD8^+^ T cells (* *p* < 0.05) over that seen in sensitized-WT mice (Figure 1E). Therefore, CD4^+^ T cells are likely involved only in exacerbating Pd allergy, because IFN-γ production by CD4^+^ T cells increased in sensitized-WT mice. However, while the ears of sensitized-I-A^b−/−^ mice and sensitized-WT mice were swollen, those of sensitized-B2m^−/−^ mice were not (Figure 2E). In addition, CD8^+^ T cells were significantly increased in sensitized-I-A^b−/−^ mice without the cooperation of CD4^+^ T cell activation (Figure 2F). These results indicate that Pd allergy is caused by antigen presentation by MHC I to CD8^+^ T cells; however, Pd allergy does not occur following the MHC II-CD4^+^ T cell interaction. Thus, both MHC I expression and activation of CD8^+^ T cells are essential for the development of Pd allergy. Consistent with our results, CD8^+^ T cells have been shown to function as effector cells in DTH responses [14].

It has been reported that PdCl_2_ is sufficiently reliable reagent for development of Pd allergy and that solution of PdCl_2_ injection induces T cell dependent allergic diseases and in mice [6,15,16]. Therefore, we used PdCl_2_ for these experiments and referred the appropriate dose of PdCl_2_ [6,15,16]. Interestingly, since Na_2_[PdCl_4_] has been shown to be effective in human patch testing [17], Na_2_[PdCl_4_] may be more useful reagent than PdCl_2_ in Pd allergic mouse model. Future experiments will be required for applying animal experiments. 

LPS is a component of microbes that, when recognized by immune cells, induces MHC expression on APCs (Figure S3B). Thus, it has been suggested that this microbial component plays a role in the immunological response leading to metal allergy [18]. Moreover, it is likely that the resident flora contributes to the progression of metal allergy.

The predominant TCRα chain expressed during Pd allergy is TRAV7-2*02/TRAJ22*01. While TRAV7-2*02 was expressed on both subsets of T cells (Figure 2C,D), increased frequencies of TRAV7-2*02 expressing CD8^+^ T cells were observed in sensitized-I-A^b−/−^ allergic mice (data not shown). Therefore, adoptive transfer experiments were performed to assess whether TRAV7-2*02 expressing CD8^+^ T cells are important for Pd allergy. Interestingly, we found ear swelling and increases in CD8^+^ T cells in mice given Pd-APCs at a similar level to conventional Pd allergic mice (Figure 3A,B). Furthermore, the TRAV7-2*02 repertoire was found to be significantly represented in mice given Pd-APCs after Pd challenge (Figure 3C). In contrast, the frequency of TRAV7-2*02 was not increased in mice given B2m^−/−^ Pd-APCs compared to mice given WT Pd-APCs (Figure 4C). Consistent with the lack of a response by TRAV7-2*02 expressing CD8^+^ T cells, Pd allergy did not develop in mice given B2m^−/−^ Pd-APCs (Figure 4). Therefore, these results indicate that CD8^+^ T cells play a role as pathogenic T cells for the development of Pd allergy, and that TRAV7-2*02 expressing CD8^+^ T cells are activated by Pd-stimulated APCs expressing MHC I. Accordingly, our findings indicate the presence of a specific TCR in metal allergy. Furthermore, the adoptive transfer experiments indicate that APCs stimulated in vitro with Pd function to sensitize recipient naïve mice. Therefore, it is possible that adoptive transfer experiments may be useful for further study of antigen presentation in metal allergy.

The skewed TCRs identified in Pd allergy were TRAV7-2*02/TRAJ22*01. As for the TCRβ chain, the TRBV13 family has been reported to be frequently expressed in various mouse disease models including in NOD mice [19] and in an induced arthritis model [20]. These results imply that TCRβ chains exhibit flexible binding capacities against pathogenic antigens. However, in Pd allergy there was actually more frequent skewing of the TCRα chain than of the TCRβ chain, and only Pd-specific TCRα chains were detected. Consistent with our results, TRAV has a high specificity for recognizing nickel bound to peptide in humans [21,22]. Therefore, the Pd binding peptide may be restricted to the TCRα chain. Moreover, we found that TRAJ22*01 was the most commonly-expressed J segment (Table 1), and we identified the CDR3 consensus frame, CAAXSGSWQLIF, in TRAV7-2*02/TRAJ22*01 (Table 2). Taken together, it can be concluded that TRAV7-2*02/TRAJ22*01 expressing CD8^+^ T cells function as pathogenic T cells in Pd allergy. Based on our results, transgenic mice expressing TRAV7-2*02/TRAJ22*01 on CD8^+^ T cells should be highly-sensitive Pd allergy animal models and would be a valuable tool to explore the molecular mechanisms underlying the pathogenesis of metal allergy. Furthermore, the response of TRAV7-2*02 expressing CD8^+^ T cells could be assessed as an indicator of metal safety and, thus, our findings may be helpful for development of innovative biomaterials.

## 4. Materials and Methods

### 4.1. Ethics Statement

All mice were maintained under specific pathogen-free conditions, and used according to the guidelines of the Institutional Animal Care and Use Committee established at Tohoku University. The project identification code is 2016AcA-016 (date of approval: 25 March 2016).

### 4.2. Mice

C57BL/6 mice were obtained from CLEA Japan (Tokyo, Japan). B2m^−/−^ mice or IFN-γ^−/−^ mice were obtained from the Jackson Laboratory (Bar Harbor, ME, USA). I-A^b−/−^ mice were kindly provided by Diane Mathis (Harvard Medical School, Boston, MA, USA).

### 4.3. Antibodies and Reagents

Monoclonal antibodies (mAbs) for immunohistochemistry were purchased from BD Biosciences (San Jose, CA, USA). All mAbs for flow cytometry were purchased from BioLegend (San Diego, CA, USA). LPS from *Escherichia coli* (O55:B5) [15,23], phorbol myristate acetate (PMA), ionomycin, and propidium iodide (PI) were purchased from Sigma-Aldrich (St. Louis, MO, USA). PdCl_2_ was purchased from Wako Pure Chemical Industries, Ltd. (Osaka, Japan).

### 4.4. Induction of Pd Allergy

Induction of Pd allergy was previously described [6]. Briefly, for sensitization mice were injected twice intraperitoneally with 250 μL of 10 mM PdCl_2_ containing 10 μg/mL LPS in PBS at an interval of seven days. LPS was used as an adjuvant [15]. As a control, mice were injected with PBS only (unsensitized). Seven days after the second sensitization, mice were challenged by intradermal injection of 20 μL of 0.5 mM PdCl_2_ into each ear. Ear swelling was measured before and after the challenge using a Peacock dial thickness gauge (Ozaki MFG Co., Ltd., Tokyo, Japan) [6,15,23,24].

### 4.5. Histological Analysis

To identify the T cells present in ear auricles of WT mice with Pd allergy, frozen ear auricles were sliced and immunostained with anti-mouse CD4 mAb (H129.19), and anti-mouse CD8a mAb (53-6.7). CD4^+^ and CD8^+^ T cells were visualized by staining with 3,3′-diaminobenzidine (DAB) chromogen, and these sections were counter-stained with hematoxylin. The DAB signals were detected with an Olympus IX81 microscope, an Olympus DP71 CCD camera (Olympus, Tokyo, Japan), and Lumina Vision software (Mitani Corporation, Fukui, Japan). The scale bar indicates 100 μm.

### 4.6. Adoptive Transfer of Bone Marrow-Derived Antigen-Presenting Cells

To differentiate APCs, mouse bone marrow cells were cultured in RPMI1640 complete medium with 10% FBS and 10% CMG14-12 culture supernatant containing mM-CSF [25,26]. On day 7, the collected APCs were treated with 0.2 mM PdCl_2_ and 10 ng/mL LPS (Pd-APCs) or 10 ng/mL LPS (LPS-APCs, as a control), and were cultured for 24 h. On day 8, these APCs were washed twice in PBS to completely remove the supernatant, and the harvested Pd-APCs or LPS-APCs (1 × 10^6^ cells per mouse) were adoptively transferred via intravenous injection into naïve C57BL/6 mice for sensitization (Figure S4). Seven days after the adoptive transfer, recipient mice were challenged by intradermal injection using the same procedure described above.

### 4.7. Flow Cytometric Analysis of Cell Populations

SLN cells were pretreated with anti-CD16 and CD32 mAbs (2.4G2) to block Fc receptors, and then stained with the following specific mAbs: anti-CD3ε (145-2C11), anti-CD4 (GK1.5), anti-CD8a (53-6.7), anti-F4/80 (CI:A3-1), anti-CD11b (M1/70), anti-CD80 (16-10A1), anti-CD86 (GL-1), anti-CD40 (3/23), anti-H-2K^b^ (AF6-88.5), and isotype-matched controls. SLN cells were washed twice, and were then stained with PI followed by analysis on a FACSCanto II (BD Biosciences). Live cells were identified based on characteristic forward and side scatter and by exclusion of PI. Cell number was calculated using flow cytometric analysis. Number of cells in each subset of T cells was calculated by the following procedure: CD3^+^ T cell numbers = total SLN cells numbers × CD3ε positive T cells population (%), and CD8^+^ (or CD4^+^) T cell numbers = total SLN cells numbers × CD3ε and CD8a (or CD4) double-positive T cell population (%). To identify IFN-γ producing T cells, SLN cells were isolated 24 hours after Pd challenge, and then were incubated with 20 ng/mL PMA plus 0.5 μg/mL ionomycin for four hours. Cells were then stained with anti-CD4, anti-CD8a, and anti-IFN-γ (XMG1.2) mAbs according to manufacturer’s instructions and analyzed using a FACSCanto II.

### 4.8. T Cell Repertoire Sequence Analysis Using a Next-Generation Sequencer

To investigate the skewing of TCRs in Pd allergy, we analyzed the TCR repertoire using a next generation sequencer. Briefly, total RNA was prepared from the SLN 24 hours after Pd challenge [16]. Complementary DNA was synthesized from total RNA and TCR chains were amplified using adaptor ligation-mediated PCR. The specific PCR primers were 5′-AGG TGA AGC TTG TCT GGT TGC TC-3′ (TCRα) and 5′-TGC AAT CTC TGC TTT TGA TGG CTC-3′ (TCRβ). Then, using the PCR products as templates, TCR sequences were analyzed using the 454 GS junior+ system (Roche Applied Science, Indianapolis, IN, USA) according to the manufacturer’s protocol. Alignments among approximately 100,000 sequences/run were performed with IMGT/V-QUEST (http://www.imgt.org).

### 4.9. Statistics

Student’s *t*-test was used for analysis of differences and values of *p* < 0.05 or 0.01 were considered statistically significant [6,27].

## 5. Conclusions

In conclusion, we show that MHC I-dependent CD8^+^ T cells activate and proliferate as pathogenic T cells and we identified TRAV7-2*02/TRAJ22*01 as the specific TCR in Pd allergic mice. Furthermore, Pd-APCs prepared in vitro can function as memory APCs and activate pathogenic T cells in recipient mice. In addition, the CDR3 consensus frame of pathogenic TCRs is CAAXSGSWQLIF in TRAV7-2*02/TRAJ22*01. Thus, these specific TCRs are promising, novel targets for generating improved diagnostics and treatments for Pd allergy.

## Figures and Tables

**Table 1:** Frequency of TRAJ repertoires bearing TRAV7-2*02.

TRAV7-2*02 Bearing TRAJ	Frequency (%)
TRAJ6*01	6.8
TRAJ15*01	10.6
TRAJ22*01	25.5
TRAJ26*01	4.6
TRAJ27*01	4.8
TRAJ31*01	4.6
TRAJ37*01	7.1

Representative data are shown. Similar results were observed in other sensitized WT mice (*n* = 4).

**Table 2:** The CDR3 amino acid sequences of TRAV7-2*02/TRAJ22*01 in Pd sensitized-WT mice.

	TRAV	CDR3			TRAJ	Frequency (%)
		3′ V-Region	N	5′ J-Region		
Mouse 1	TRAV7-2*02	CAAR	G	SGSWQLIF	TRAJ22*01	22.1
	TRAV7-2*02	CAA	T	SGSWQLIF	TRAJ22*01	20.1
	TRAV7-2*02	CA	AGA	SSGSWQLIF	TRAJ22*01	20.1
	TRAV7-2*02	CAAS	I	SGSWQLIF	TRAJ22*01	14.9
	TRAV7-2*02	CA	AA	SSGSWQLIF	TRAJ22*01	14.9
	TRAV7-2*02	CA	ARA	SSGSWQLIF	TRAJ22*01	7.8
Mouse 2	TRAV7-2*02	CAA	SHA	SSGSWQLIF	TRAJ22*01	39.3
	TRAV7-2*02	CA	AR	SSGSWQLIF	TRAJ22*01	39.3
	TRAV7-2*02	CAA	IA	SSGSWQLIF	TRAJ22*01	21.4
Mouse 3	TRAV7-2*02	CA	AR	SSGSWQLIF	TRAJ22*01	69.0
	TRAV7-2*02	CAA	KIP	GSWQLIF	TRAJ22*01	31.0
Mouse 4	TRAV7-2*02	CA	AA	SSGSWQLIF	TRAJ22*01	33.3
	TRAV7-2*02	CAAS	L	SSGSWQLIF	TRAJ22*01	42.8
	TRAV7-2*02	CAAS	P	SSGSWQLIF	TRAJ22*01	23.9

We identified a consensus frame, CAAXSGSWQLIF (*X* = 1 to 4 amino acids) (*n* = 4).

**Table 3:** The CDR3 amino acid sequences of TRAV7-2*02/TRAJ22*01 in mice given WT Pd-APCs.

	TRAV	CDR3			TRAJ	Frequency (%)
		3′ V-Region	N	5′ J-Region		
Mouse 1	TRAV7-2*02	CAA	T	SSGSWQLIF	TRAJ22*01	30.8
	TRAV7-2*02	CAA	S	SGSWQLIF	TRAJ22*01	23.1
	TRAV7-2*02	CAAS	PA	SSGSWQLIF	TRAJ22*01	15.4
	TRAV7-2*02	CAA	R	SSGSWQLIF	TRAJ22*01	15.4
	TRAV7-2*02	CA	AR	SGSWQLIF	TRAJ22*01	15.4
Mouse 2	TRAV7-2*02	CA	AA	SSGSWQLIF	TRAJ22*01	30.8
	TRAV7-2*02	CAAS	A	SGSWQLIF	TRAJ22*01	23.1
	TRAV7-2*02	CAAS	R	SSGSWQLIF	TRAJ22*01	15.4
	TRAV7-2*02	CA	AR	SSGSWQLIF	TRAJ22*01	15.4
	TRAV7-2*02	CA	AQ	SSGSWQLIF	TRAJ22*01	15.4
Mouse 3	TRAV7-2*02	CAAS	I	SSGSWQLIF	TRAJ22*01	50.0
	TRAV7-2*02	CAAS	I	SGSWQLIF	TRAJ22*01	50.0
Mouse 4	TRAV7-2*02	CAA	T	SSGSWQLIF	TRAJ22*01	25.9
	TRAV7-2*02	CAAS	MA	SGSWQLIF	TRAJ22*01	25.9
	TRAV7-2*02	CAAS	I	SSGSWQLIF	TRAJ22*01	18.5
	TRAV7-2*02	CAA	GS	SSGSWQLIF	TRAJ22*01	14.8
	TRAV7-2*02	CAA	SP	SGSWQLIF	TRAJ22*01	14.8

Similarly, the CDR3 amino acid sequences of TRAV7-2*02/TRAJ22*01 are shown (*n* = 4).

## Supplementary Materials

Supplementary materials can be found at www.mdpi.com/1422-0067/18/6/1162/s1.

Supplementary File 1

Supplementary File 2

## Abbreviations

Pd	Palladium
DTH	Delayed-type hypersensitivity
TCR	T cell receptor
TRAV	TCRα variable
APCs	Antigen presenting cells
MHC	Major histocompatibility complex
TRAJ	TCRα junction
WT	Wild-type
SLN	Submandibular lymph node
LPS	Lipopolysaccharide
PBS	Phosphate buffered saline
SD	Standard deviation
TRBV	TCRβ variable
B2m	β2m-Microgloblin
B2m^−/−^ mice	C57BL/6 deficient β2m-microgloblin mice
I-A^b−/−^ mice	C57BL/6 deficient MHC II *H2-Ab1* (I-A^b^) mice
mM-CSF	Mouse macrophage colony stimulating factor
mAbs	Monoclonal antibodies
PMA	Phorbol myristate acetate
PI	Propidium iodide
