# Supplementary figures and images for: TRAV7-2*02 Expressing CD8+ T Cells Are Responsible for Palladium Allergy
Source: Int J Mol Sci. 2017 May 31;18(6):1162. doi: 10.3390/ijms18061162 (PMC5485986; doi:10.3390/ijms18061162)

Supplemental materials

Figure S1, A

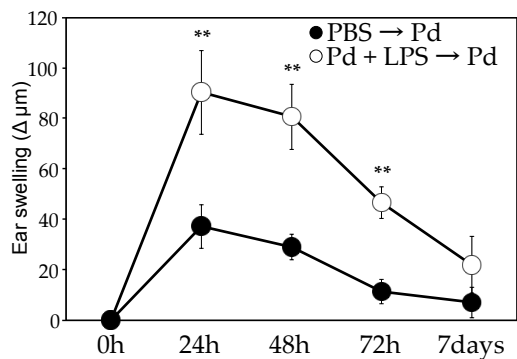

Figure S1, B

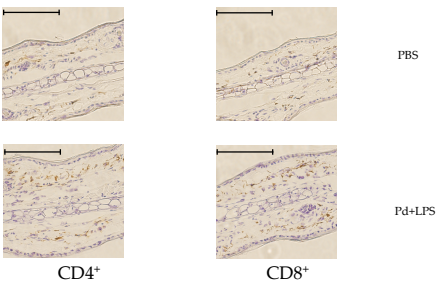

Figure S2

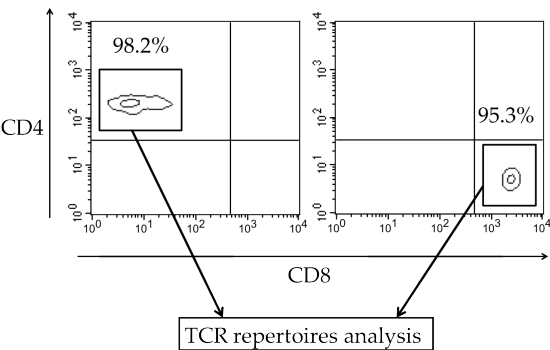

Figure S3, A

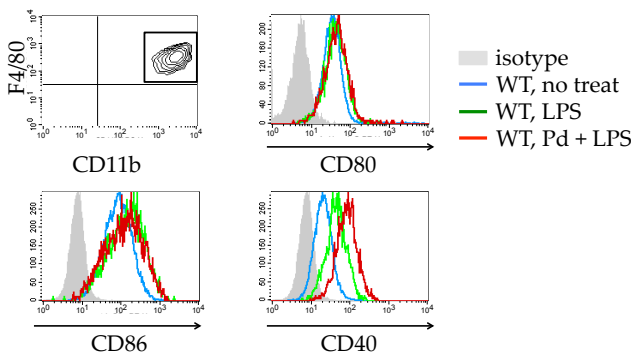

Figure S3, B

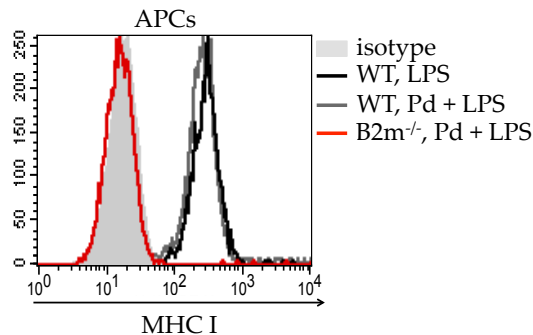

Figure S4

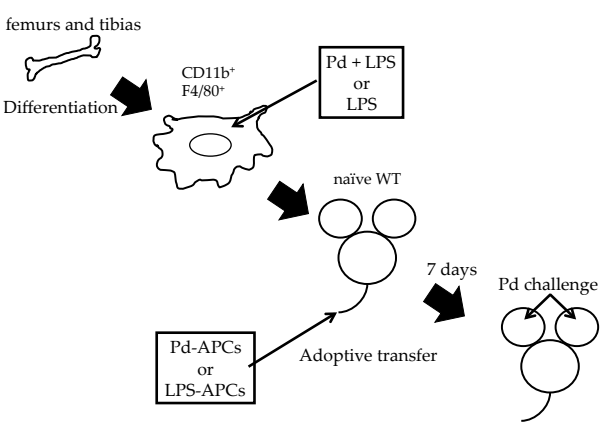

Supplement: Supplementary File 1 [file ijms-18-01162-s001.pdf]
