# Supplementary material for: TRAV7-2*02 Expressing CD8+ T Cells Are Responsible for Palladium Allergy
Source: Int J Mol Sci. 2017 May 31;18(6):1162. doi: 10.3390/ijms18061162 (PMC5485986; doi:10.3390/ijms18061162)
Supplement: Supplementary File 2 [file ijms-18-01162-s002.pdf]

Supplemental materials

Figure S1. Induction of Pd allergy in C57BL/6 mice.

(A) Ear swelling in Pd allergy-induced WT mice (n = 5). Values are means ± SDs. $^{**}P < 0.01$. Similar results were obtained in two independent experiments. (B) In Pd allergy-induced WT mice, $CD4^+$ and $CD8^+$ T cells in ear auricles were visualized by DAB, and these sections were counterstained with hematoxylin. Scale bar indicates 100 μm.

Figure S2. Isolation of $CD8^+$ T cells and $CD4^+$ T cells from SLN.

SLN cells were isolated at 24 hours after Pd challenge, and $CD4^+$ T cells and $CD8^+$ T cells were isolated using anti-CD4 mAb conjugated MACS® beads (Miltenyi Biotec, Bergisch Gladbach, Germany) in unsensitized-WT mice and sensitized-WT mice (n = 5). Presence of $CD8^+$ T cells (> 95%) or $CD4^+$ T cells (> 95%) was measured by flow cytometry, and the TCR repertoire was analyzed using a next generation sequencer.

Figure S3. Cell surface marker analysis of bone marrow-derived APCs.

(A) Untreated WT APCs, WT Pd-APCs, and WT LPS-APCs were stained with anti-F4/80 (CI:A3-1), anti-CD11b (M1/70), anti-CD80 (16-10A1), anti-CD86 (GL-1), anti-CD40 (3/23), and isotype-matched control mAbs and were examined by flow cytometry. (B) Using an anti-H-$2K^b$ (AF6-88.5), expression of MHC class I was compared among WT Pd-APCs, WT LPS-APCs, and $B2m^{-/-}$ Pd-APCs.

Figure S4. Illustration of APC adoptive transfer

$CD11b^+$ and $F4/80^+$ APCs from WT or $B2m^{-/-}$ mice were cultured with mM-CSF and treated with Pd + LPS or LPS and were then transferred to naïve WT mice.
